# Adult capped dental payment model applied within a university setting: an Australian reflective case study

**DOI:** 10.1186/s12903-021-01774-y

**Published:** 2021-08-23

**Authors:** Jennifer Hanthorn Conquest, John Skinner, Estie Kruger, Marc Tennant

**Affiliations:** 1 School of Human Sciences, Faculty of Science, The University of Western Australia (M309), 35 Stirling Highway, Perth, WA 6009 Australia; 2 Poche Centre for Indigenous Health, Edward Ford Building (A27), The University of Sydney, Sydney, NSW 2006 Australia

**Keywords:** Dental students, Capitation, Rural and remote dental services

## Abstract

**Background:**

Capitation models of care in dentistry started around 1973 with varying degrees of success in meeting the needs of the individuals and expectations of the participating private practitioners. These studies mostly identified that capitation payments resulted in under treatment whilst fee-for-service models often led to over treatment. The objective of this study was to develop a new way of doing business using an outsourcing capitation model of care to meet population health needs and activity-based funding requirements of rural Local Health Districts with a local university dental school. This payment model is an alternate referral pathway for public oral health practitioners from the existing New South Wales Oral Health Fee-for-Service Scheme that focuses on urgent treatment to one that offers an all-inclusive preventive approach that concentrates on sustaining good long-term oral health for the individual.

**Method:**

The reflective study analysed various adult age cohorts (18–24, 25–34, 35–44, 45–54, 55–64, 65–74 and 75 + years) based on 950 participants randomly selected from the Greater Southern adult public dental waiting lists. The study’s capitation formula was derived from NSW government adult treatment items (n = 447,625). Dental care was provided through the local university’s dental clinics utilising only dental students under clinical supervision. All data were sourced from NSW Oral Health Data Warehouse during 1 January 2012–30 June 2018 and analysed by using SAS 9.3 and Version 13 Microsoft Excel.

**Results:**

There were 10,305 dental care items and 1129 capitation courses of care totalling A$599,026. This resulted in an average of 11 dental care items being provided to each participant. The capitation payment formula utilising the most provided dental care items of 100 individual patients proved to be economical and preventive focused.

**Conclusion:**

The systematic reflection showed that this unique methodology in developing an adult capitation payment formula associated to diagnostic pathways that resulted in: (i) more efficient usage of government expenditure on public dental services, (ii) provision of person-centred courses of dental care, and (iii) utilisation of university dental education programs to best practice treatment and holistic care.

## Background

Over the past 20 years, the Commonwealth government in Australia has funded the development of rural-based universities (and in the case of dentistry—dental schools), in major regional centres to encourage more dentists to live and work rurally [1, 2]. As a by-product of this move to re-distribute dental education, residents adjacent to these dental schools now have access to government-subsidised, student-provided dental care, supervised by the University [3].

In 2013 and 2014 the Commonwealth government initiated the Voluntary Dental Graduate Year Program and the Oral Health Therapist Graduate Year Program, respectively. These programs were structured to enhance the practical experience of the student, as well as increasing the work capacity whilst being mentored in clinical settings within the public dental system and other areas of need such as Aboriginal Community Controlled Health Services [4].

In 2002 it was shownby Richards et al. (2002) that dental care provided by undergraduate students was 50–70% of the cost of care provided by public dentists [5].

There have been several New South Wales (NSW, the most populated State of Australia)) strategies to reduce the service gaps for rural and remote Australians, such as ‘growing our own’ supply of dental professionals [6]. Another is to implement the ‘NSW Oral Health Plan 2020’ that involves working with “Primary and Tertiary Service Delivery”, and supports the implementation of [7]:

1. “Identify and address inadequacies in existing models of care and incorporate an evidence-based preventive and therapeutic approach to service provision, including timely dental assessments and provision of individual oral health care.”
2. “Develop and implement initiatives that encourage greater participation by private sector dentists and other organisations in the treatment of public clients.” [8]

The report of the National Advisory Council on Health (2012) agreed that “the long‐term goal for dental health in Australia should be a system that allows universal access to dental care.” [9] However, only four options were provided within a preparatory framework with the first and third being capped fee entitlements.

To access these entitlements (for all ages), they would be required to hold a government concession card [9]. These entitlements would be provided through the Medicare Chronic Disease Dental Scheme and Medicare Teen Dental Program [9].

In 2012 the Commonwealth introduced a Dental National Partnership Agreement (DNPA) for all Australian States and Territories with substantial additional funding for adult dental care [10]. This DNPA introduced weighted activity units to measure the services’ output to ensure increased access and free treatment [10].

The DNPAs also required substantial delivery of additional services above historical levels. Many states had limited infrastructure to deliver the required rapid increases in service provision, and this led to testing various new procurement models-of-care.

The constraints on the public dental system resulted in focusing on emergency care with a high percentage of individuals receiving extractions [11]. With the DNPA now placing greater pressure on the public dental service to meet activity targets, there will be less inclination to provide preventive dental care [11].

The implementation of the DNPA and associated activity measures provided an opportunity to benchmark the capitation study to existing NSW dental service models and provide full courses of dental care. This benchmarking between government services, fee-for-service and capitation is based on identical treatment item price [12].

The specific objectives of this study were to analyse the treatment provided, during the period 2012/13 to 2017/18 through three cost comparisons: (i) in-house public dental services (state government clinics); (ii) State government dental fee-for-service scheme (public dental vouchers in private dental clinics); and (iii) the study’s capitation payments.

The study’s hypothesis was aimed at determining that the CMoC formula developed on dmft/DMFT and weighted treatment items (of specific age groups), will be an economic payment scheme that does not result in underservicing.

## Methods

All data was sourced from NSW Oral Health Data Warehouse during 1 January 2012–30 June 2018 and analysed by using SAS 9.3 and Version 13 Microsoft Excel.

This study examined the capitation formula, based on NSW government adult treatment items (n = 447,625). The treatment items were ranked according to the largest number of treatments that were delivered (Table 1), providing the basis for the capitation diagnostic pathways formula.

**Table 1:** Capitation formula by age groups for each of the diagnostic pathways

Diagnostic pathway	Weighting								
	Removal of calculus subsequent visit (115)	Fluoride treatment (123)	Extraction (311)	2 Surface anterior restoration (512)	1 Surface anterior restoration (521)	2 Surface anterior restoration (522)	1 Surface posterior restoration (531)	2 Surface posterior restoration (532)	Temporary restoration (572)
No active caries no pain									
18 − < 25 years	–	–	–	–	–	–	–	–	–
25 − < 35 years	–	1	–	–	–	–	1.87	–	–
35 − < 45 years	–	–	–	–	–	–	1.83	1.39	–
45 − < 55 years	–	–	–	–	–	–	–	–	–
55 − < 65 years	–	–	–	–	–	–	–	–	–
65 − < 75 years	–	–	–	–	–	–	1.95	–	–
75 + years	–	–	–	–	–	–	–	–	–
Active caries no pain									
18 − < 25 years	–	–	–	–	–	–	1.95	–	–
25 − < 35 years	–	–	2	–	1.67	–	2	–	–
35 − < 45 years	–	–	2.74	1.5	1.85	–	1.78	1.35	1.7
45 − < 55 years	1	–	2.68	–	1.86	–	1.85	1.27	–
55 − < 65 years	–	–	2.17	–	1.98	–	1.88	1.1	–
65 − < 75 years	1	–	2.02	–	2.15	1.47	1.88	1.23	–
75 + years	–	–	1.97	–	2.2	–	1.7	–	–
Active caries no pain									
18 − < 25 years	–	–	1.5	–	–	–	1.85	–	–
25 − < 35 years	–	–	1.83	–	1.62	1.47	1.9	1.39	1.57
35 − < 45 years	–	1	2.07	1.44	1.72	1.45	1.81	1.25	1.6
45 − < 55 years	–	–	2.37	–	1.81	1.45	1.72	1.22	–
55 − < 65 years	–	–	2.17	–	1.86	1.44	1.73	1.1	–
65 − < 75 years	–	–	2.14	–	2.11	1.4	1.66	1.19	–
75 + years	–	–	2.03	–	2.11	1.41	1.56	1.18	–

The weighting for each of the treatment items, for each of the age groups, displayed in Table 1 was developed from the following equation:

### Total number of treatment items / total number of NSW distinct individuals who were provided the items) × 100 = item weighting

The items identified with the weighting of one (1), determined as an essential, were given a full weighting, while the items with a weighting of zero (0) were expected to be provided but not paid for. For example, the items “full examination” (011) and “oral hygiene instruction” (141) were given a weighting of zero for all age groups. Whilst “radiographs” (022) and “removal of calculus first visit” (114) were weighted as one (1) for all ages.

The data analysis was based on the different aspects of the study: (i) de-identified adult data grouped into age bands (as below); and (ii) capitation diagnostic pathways, periodontal course of care and additional treatment items that were paid at an extra cost.

The capitation diagnostic pathways were divided into: (i) “No active caries and no pain”—dental categories of diagnostic, preventive, and restorative; (ii) “Active caries and no pain”—dental categories of diagnostic, preventive, restorative and exodontia; and (iii) “Active caries and pain”—dental categories of diagnostic, preventive, restorative and exodontia. All diagnostic pathways included removal of calculus and prophylaxis treatment.

The “Periodontal Course of Care” provided for clinical periodontal analysis, treatment for acute infections, and periodontal debridement. “Additional treatment items” were offered based on their complexity such as root canal therapy, surgical removal of a tooth, periodontal flap surgery, pin retention, metallic crown, cups capping and splinting and stabilisation.

The monetary weighting of each treatment item was based on the relative pricing used by the DNPA for government public dental services and those used by the State Oral Health Fee-for-Service Scheme (OHFFSS). The OHFFSS fee schedule remained unchanged until 2013–2014 when a 2% increase was provided. The fees increased by a further 2% in 2018 [13]. The capitation monetary weighting for each item was at the 2011 DNPA’s pricing and remained unchanged throughout the study, with the NSW median cost for an individual course of care was A$590.00.

The university capitation payments for each of the age groups and diagnostic pathways were:

- 18–24 years—no active caries, no pain (A$100.25), active caries, no pain (A$253.65), active caries and pain (A$371.40)
- 25–34 years—no active caries, no pain (A$264.60), active caries, no pain (A$548.05), active caries and pain (A$839.75)
- 35–44 years—no active caries, no pain (A$281.45), active caries, no pain (A$914.40), active caries and pain (A$980.10)
- 45–54 years—no active caries, no pain (A$100.25), active caries, no pain (A$769.80), active caries and pain (A$817.45)
- 55–64 years—no active caries, no pain (A$100.25), active caries, no pain (A$684.50), active caries and pain (A$792.40)
- 65–74 years—no active caries, no pain (A$253.65), active caries, no pain (A$865.75), active caries and pain (A$808.10)
- 75 + years—no active caries, no pain (A$100.25), active caries, no pain (A$561.00), active caries and pain (A$790.95)

The administrative payment processes varied in the different university locations, and this resulted in not all periodontal courses of care, or additional treatment items, being paid for at an additional cost to the capitation diagnostic pathways. Thus, some capitation payments were not consistent with the associated fee-schedules. The participation rate in the study during 2016/17 to 2017/18 was low as the SLA ended on 30 April 2016, was considered statistically insignificant and therefore excluded.

## Results

The study’s results were divided into two statistical categories, these being: (i) the total pricing for the three payment models and the treatment provided (2012/13 to 2017/18); and (ii) the 3 payment models, treatment provided and age group during 2012/13 to 2015/16.

### Cost comparison

The overarching comparison between the government in-house dental services, OHFFSS services and capitation, resulted in the study’s trial focusing on the most cost-efficient payment model.

**Fig. 1:**
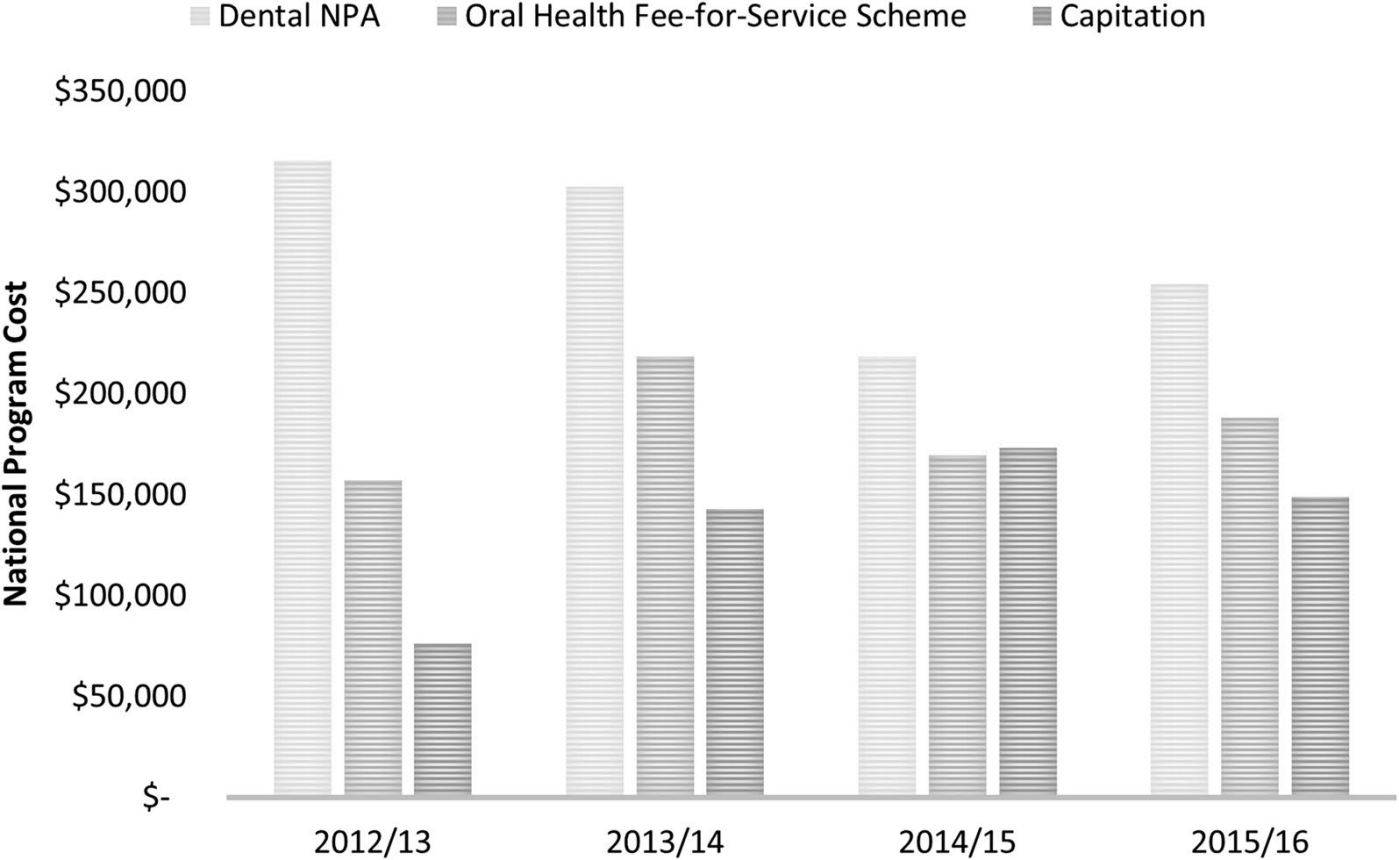
Comparison between DNPA, Oral Health Fee-for-Service Scheme and Capitation, 2012/13–2015/16

In total, the DNPA cost for Greater Western NSW for the study period 2012/13 to 2017/18 was A$1,126,157. Figure 1 shows the analysis for the period between 2012/13 to 2015/16 in which time the DNPA cost was A$1,090,586, with the OHFFSS cost at 67% (A$732,916) and capitation 50% of the DNPA (A$541,270).

### Capitation study

The study provided 950 participants with access to treatment within the universities’ dental clinics during 2012/2013 to 2017/18. Students provided 950 diagnostic and 151 periodontal courses of care as well as 82 additional treatment items.

The breakdown of the participants’ outcomes during 2012/13–2015/16 (Table 2) was for the diagnostic pathways: (i) 208 “no active caries and no pain” courses of care; (ii) 596 courses of care for “active caries and no pain”, and (iii) 92 “active caries and pain” courses of care (Table 3).

**Table 2:** Capitation cost and associated number of treatment items, 2012/13–2015/16

Year	Diagnostic pathways	Diagnostic$	Periodontal care	Periodontal care$	Additional items	Additional items$
2012/13	99	71,086.00	26	3500.00	24	1434.00
2013/14	227	13,7043.00	35	4711.00	21	1271.00
2014/15	264	166,804.00	35	4711.00	18	1817.00
2015/16	221	143,935.00	30	4038.00	13	921.00
2016/17	82	52,569.00	24	3230.00	6	336.00
2017/18	3	1486.00	1	135.00	0	0.00
Total	896	572,924.00	151	20,325.00	82	5779.00

**Table 3:** Number of diagnostic pathways, periodontal care and additional treatment items payment vouchers, 2012/13–2015/16

Age group (years)	Diagnostic pathways			Periodontal Care	Additional items
	No active caries No pain	Active caries No pain	Active caries Pain		
18–24	86	38	66	8	5
25–34	6	90	13	21	13
35–44	0	97	2	20	7
45–54	11	74	5	25	12
55–64	105	6	3	28	16
65–74	0	204	2	35	26
75 +	0	87	1	14	3
Total	208	596	92	151	82

There was a total of 10 305 treatment items provided, averaging 11 items per person. The top ten (10) treatment items for the combined age groups were: (i) single x-rays (n = 1802–17.5%); (ii) adhesive restoration on a single surface of a posterior tooth (n = 938–9.1%); (iii) full diagnostic examination (n = 841–8.2%); (iv) removal of calculus (n = 764–7.4%); (v) removal of a tooth (n = 706–6.9%); (vi) adhesive restoration on a single surface of an anterior tooth (n = 679–6.6%); (vii) dental education (n = 540–5.2%); (viii) adhesive restoration on two (2) surfaces of a posterior tooth (n = 341–3.3%); (ix) sealing of a non-carious lesion (n = 323–3.1%); and (x) periodontal treatment per tooth (n = 322–3.1%).

### Age group analysis

The age groups (2012/13–2017/18) which provided the most treatment items and access to the students through the capitation pathways in order were: 65–74 years (n = 2206), 18–24 years (n = 1912), 25–34 years (n = 1549), 55–64 years (n = 1467), 35–44 years (n = 1239), 45–54 years (n = 1040) and 75 + years (n = 892).

**Fig. 2:**
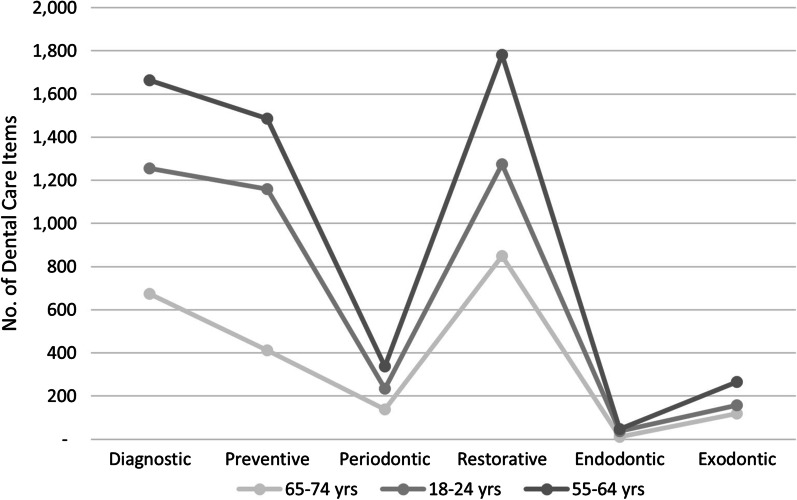
Age Group Comparison of Dental Care Categories Provided, 2012/13–2015/16

The age groups that had the most access to the capitation pathways were 65–74 years, 18–24 years, and 55–64 years age groups (Fig. 2).

### 65–74 years age group

The 65–74 years age group were provided 206 diagnostic pathways with 204 “active caries and no pain”, 2 “active caries and pain”, and 35 periodontal courses of care.

The total number of treatment items provided was 2206 which included 26 payments for additional items that included endodontics (n = 9), exodontia (n = 6) and restorative treatment components such as pin retention and metallic crowns (n = 41).

In this age group, the total cost comparison is as follows: government in-house service cost (A$225,646), OHFFSS (A$173,549) and capitation (A$178,229).

The capitation payments for the diagnostic pathways were active caries, and no pain (A$176,613) and active caries and pain (A$1616) with the diagnostic pathway mean at A$865.

### 18–24 years age group

The 18–24 years age group were provided 86 “no active caries and no pain”, 38 “active caries and no pain” and 66 “active caries and pain”, and 8 periodontal courses of care.

The total number of treatment items provided was 1912, which included 5 payments for additional items that included exodontia (n = 1) and endodontics (n = 18).

The total cost comparison for this age group was government in-house service (A$148,828); OHFFSS (A$111,977) and capitation (A$52,472).

The capitation payments for “no active caries and no pain” was A$15,971, “active caries and no pain” (A$10,572) and “active caries and pain” (A$24,512), with the diagnostic pathway mean at A$268.

### 55–64 years age group

The age group from 55 to 64 years were provided 105 “no active caries and no pain”, 6 “active caries and no pain” and 3 “active caries and pain”, as well as 28 periodontal courses of care.

The total number of treatment items provided was 1467, which included 16 payments for additional items that included endodontics (n = 9), exodontia (n = 4), additional periodontal care (non-surgical treatment) (n = 10) and periodontal maintenance (n = 7).

The total cost comparison for this age group was as follows: government in-house service (A$150,940); OHFFSS (A$113,140) and capitation (A$79,824).

The capitation payments for “no active caries and no pain” was A$72 128, “active caries and no pain” (A$4107) and “active caries and pain” (A$3337), with the diagnostic pathway mean at A$690.

## Discussion

Capitation payment programs have been researched in the private practitioner setting, particularly in the United Kingdom and Scandinavia. Studies in Norway, United States of America Sweden, and Scandinavia have examined likes, dislikes and the risk impact on the type of dental care provided such as fee-for-service (over-servicing) and capitation (under-servicing or no-change in care provided) [14, 15].

Atchison and Schoen’s (1990) study shows that fee-for-service payment is the preferred option by the private practitioner, rather than capitation payments [23]. The consumer’s perspective as presented by Andås and Hakeberg (2014), stated that their preference is the capped prepayment fee [16, 17]. A review of a pilot contract capitation system found that contracted services provided fewer restorations, but also less prevention [15].

The authors noted that often there are individual differences between the two models-of-care which make comparisons difficult, but capitation practitioners may be better at establishing rapport with an individual, which helps promote good dental health behaviours [18]. They also found that capitation is cost-effective and can be used to target areas where there is a shortage of service providers e.g., rural, and remote areas [18].

A large-scale review of 6 years of capitation versus fee-for-service data in Sweden also found that fewer restorations were provided and capitation participants, in general, maintained better oral health status over the 6-year period [19]. The present study is unique as it examines the methodology used to develop a capitation program, the impact on the participants, and students’ access to socioeconomically disadvantaged residents.

The research of Wallace and MacEntee’s (2012) discusses the concerns of private dentists’ providing care for low-income individuals to reduce inequity, may find the study’s capitation formula of interest [20]. The recent Productivity Commission Inquiry into Human Services in Australia also made several recommendations relating to improving individual choice in accessing public dental services, and payment-models that could improve effectiveness [21].

In 2012–2014, NSW undertook a dental capitation pilot of 20 case studies that provided 365 treatment items to adults aged 65 years and over. The dental care was delivered by the private sector, in the Greater Southern Health area. This adult study was the first to test the capitation fee formula and diagnostic pathways discussed in this analysis.

The pilot study identified that 60% of the case studies required further dental treatment following their full capitation course-of-care [22]. Due to the limitation of the pilot study design it could not be determined that the capitation model-of-care provided by the private sector could or could not be advantageous as an alternative public service delivery.

In this study, it also examined the type of treatment provided by the students to the capitation formula, comparing the 3 payment systems with the common factor between them being the DNPA’s weighted activity price.

It may be argued that capitation had an advantage, as the payment price was discounted by 65% and there was not a payment increase during the study. The reason for the discount was based on a previous paediatric capitation study that also utilised oral health therapy students to provide the care and that they cost less than dentists [23].

While the OHFFSS had a periodic increase during 2013–2014 and 2018. The DNPA comparisons were standardised using a common price across the study period. Moreover, Richard et al. (2002) found that “student-provided services, i.e., all travel, accommodation, supervision, management, etc., compares positively with the cost of similar treatment provided by the private practitioner” [6].

The study’s DNPA budget (2012–2018) was A$1,126,157, of which 53% was allocated to the capitation payments. If the treatment provided by the students were outsourced through the OHFFSS, 67% of the budget would have been required to pay for the same amount of dental care (Fig. 1).

In 2012–2014 study that analysed the 20 case studies it was derived that the same capitation formula was not the most cost effective [22]. Nonetheless when the capitation formula (Table 1) was analysed in this larger cohort of 950 participants, the nuances of an individual’s dental status are reduced.

Moreover, the finding of this study is supported by the Australian Institute of Health and Welfare report [24] which identified that the most common care for adults was (in order of priority) diagnostic, restorative, and preventive. The report concluded that the mean cost per private dental visit included 18–34 years $212, 65–74 years $366 and 75 + years $331, which provided an average total of 2.5 treatment items [25]. In contrast, this study concluded that the mean cost was between $268–$865 for a full course care that on average provided 11 treatment items.

The study also confirmed that the accuracy of the ratio between capitation formula per age group and treatment categories was accurate (Table 1 and Fig. 2). This result would determine when to use the capitation model-of-care, which should be considered when demand out ways supply for both metropolitan, rural, and remote areas.

Ekanayake et al., 2011 showed that the top dental care items are reflective of the study’s top 10, being treatment for decayed teeth (restorations and exodontia) [25]. However, in comparison the Ekanayake et al., 2011 study concluded that more individuals required periodontal care, and in the current study less periodontal treatments were needed [25].

With researched evidence of linkages between periodontal disease and some systemic diseases such as coronary heart disease it may be prudent that an additional capitation diagnostic pathway is researched for high-risk individuals [26]. This diagnostic pathway could be formulated on sex, medications, ethnicity, presence of systemic disease/s and smoking, oral health literacy and behaviour and socioeconomic status and level of education [27].

The Productivity Commission Report (PCR) suggested various options for greater patient choice by implementing a system-wide prospective. This may mean that in future, the Commonwealth will provide a set amount for each eligible adult patient every 2 years, and the patient then chooses the model-of-care for accessing dental services locally. This may allow for bulkbilling via a private practitioner, public clinics, or university clinics. This could be based on the Adult Dental Benefits Scheme previously suggested by the Australian Dental Association nationally [21].

A limitation of this study is that the Greater Western Area Health Service Human Research Ethics Committees did not approve the inclusion of participant satisfaction surveys. They noted that these surveys are part of public health service standard quality and accreditation processes.

Although the capitation study proved to be cost effective, feedback from the participants would provide valuable information on their perceptions of quality of care, if their oral health needs were met, and self-assessment of their dental health. Patient satisfaction surveys are recommended as part of future studies.

Therefore, the comprehensive evidence provided demonstrates that this CmoC is cost effective and does not lend itself to underservicing of dental treatment. Moreover, it has the potential to address the payment concerns of private dental practitioners and provide effective support to public oral health services in meeting their activity targets. This research should now be revised to accommodate the current pricing of the OHFFSS and DNPA prior to being implemented as government policy.

## Conclusion

The main findings of this study demonstrate that the participants received on average 11 items of care and that the mean payment for a full course of care ranged from $268–$865.

Additionally, the capitation diagnostic pathways have shown that the combination of several treatments provided, the capitation formula and the treatment item weighting based on 100 unique individuals.

This cost-effectiveness has the potential to decrease local inequities by increasing rural-remote resident’s choice for accessing public dental care via government, fee-for-service, and capitation pathways, which is in keeping with the PCR recommendations and support implementing a system wide approach.

## Data Availability

Our legally binding data use agreement with the custodian of the data does not allow public sharing as derived from public health application.

## Abbreviations

DNPA: Dental National Partnership Agreement
NSW: New South Wales
OHFFSS: Oral Health Fee for Service Scheme
PCR: Productivity Commission Report
SLA: Service level agreement
